# The COVID-19 pandemic: a global health crisis

**DOI:** 10.1152/physiolgenomics.00089.2020

**Published:** 2020-09-29

**Authors:** Casey A. Pollard, Michael P. Morran, Andrea L. Nestor-Kalinoski

**Affiliations:** ^1^Department of Surgery, The University of Toledo, College of Medicine and Life Sciences, Toledo, Ohio; ^2^The University of Toledo Advanced Microscopy and Imaging Center, The University of Toledo, College of Medicine and Life Sciences, Toledo, Ohio

**Keywords:** ARDS, COVID-19, genetics, GTPases, pulmonary fibrosis

## Abstract

The novel coronavirus SARS-CoV-2 was identified as the causative agent for a series of atypical respiratory diseases in the Hubei Province of Wuhan, China in December of 2019. The disease SARS-CoV-2, termed COVID-19, was officially declared a pandemic by the World Health Organization on March 11, 2020. SARS-CoV-2 contains a single-stranded, positive-sense RNA genome surrounded by an extracellular membrane containing a series of spike glycoproteins resembling a crown. COVID-19 infection results in diverse symptoms and morbidity depending on individual genetics, ethnicity, age, and geographic location. In severe cases, COVID-19 pathophysiology includes destruction of lung epithelial cells, thrombosis, hypercoagulation, and vascular leak leading to sepsis. These events lead to acute respiratory distress syndrome (ARDS) and subsequent pulmonary fibrosis in patients. COVID-19 risk factors include cardiovascular disease, hypertension, and diabetes, which are highly prevalent in the United States. This population has upregulation of the angiotensin converting enzyme-2 (ACE2) receptor, which is exploited by COVID-19 as the route of entry and infection. Viral envelope proteins bind to and degrade ACE2 receptors, thus preventing normal ACE2 function. COVID-19 infection causes imbalances in ACE2 and induces an inflammatory immune response, known as a cytokine storm, both of which amplify comorbidities within the host. Herein, we discuss the genetics, pathogenesis, and possible therapeutics of COVID-19 infection along with secondary complications associated with disease progression, including ARDS and pulmonary fibrosis. Understanding the mechanisms of COVID-19 infection will allow the development of vaccines or other novel therapeutic approaches to prevent transmission or reduce the severity of infection.

## CORONAVIRUSES AND SARS-CoV-2 GENETICS

Coronaviruses are a well-studied group of viruses in the *Coronaviridae* family that are known for their ability to infect a variety of hosts due to their capacity to evolve in epidemiological situations, including crossing species barriers, mutagenesis, tissue tropism, and pathogenicity (10b, 14, 83). Coronaviruses are round, enveloped virions roughly 80–220 nm in diameter that contain a single-stranded, positive-sense RNA genome of ∼26–32 kb surrounded by an extracellular membrane containing a casing of spike glycoproteins (32, 80). The term corona in Latin translates to crown and was given to these viruses due to the presence of the spike casing that resembled a “crown-like structure” using electron microscopy (37).

Coronaviruses have been implicated in human disease as early as the late 1960s, where they were identified as the causative agents in respiratory illnesses that presented with mild symptoms associated with the common cold (32). Seven strains of human coronaviruses have been characterized, four of which are known to infect the upper respiratory tract and cause mild symptoms, while the three others are known for their severe disease-causing characteristics of the lower respiratory tract including the following: SARS-CoV (severe acute respiratory syndrome), MERS-CoV (Middle East respiratory syndrome), and SARS-CoV-2 (COVID-19) (42). Since the emergence of the COVID-19 pandemic, data-sharing initiatives have led to the much needed generation of SARS-CoV-2 data, including complete reference genomes in the National Center for Biotechnology Information database (NC_045512.2), which contains the 29,903 bp genomic sequence (83).

While it is known that the RNA polymerase of viruses lack proofreading capacity, the ensuing result is a high mutation rate with low replicative fidelity. In contrast, the coronaviruses possess an exonuclease proofreading capability that has resulted in the expansion and maintenance of one of the largest known viral genomes at ∼30 kb (17, 60). The large viral genome of SARS-CoV-2 codes for four structural proteins including the envelope, membrane, nucleocapsid, and spike glycoprotein, which play a role in both molecular characterization and host cell entry (23, 35). The SARS-CoV-2 genome also includes 16 nonstructural proteins and 9 accessory proteins required for replication and pathogenesis (23, 35, 60). While SARS-CoV-2 and SARS-CoV are 75–80% identical (3, 89), SARS-CoV-2 displays the highest sequence similarities with BatCoV at 96.2% (11). Global sequence comparison of SARS-CoV-2 isolates have expanded the literature and information known for this virus in a short period of time. Initial analysis of roughly 100 genomes of SARS-CoV-2 identified two major subtypes, designated L and S, which vary due to the presence of two linked single nucleotide polymorphisms (71). Interestingly, the L subtype is a derivative of the S type and was identified in ∼70% of the genomes compared with the S type in the remaining 30% (71). Phylogenic tree analysis of the L type suggests that the differences are related to a significantly higher mutation rate, which, consequently, results in higher transmission and/or replication rates (71). Furthermore, the SARS-CoV-2 virus has geographically diverse strains that seemingly vary in severity, mortality rate, and treatment options that were characterized using phylogenetic network analysis of 160 SARS-CoV-2 genomes (18). Three distinct viral clusters (A, B, and C) were identified with derivative subgroups, with cluster A sharing the closest similarity to the BatCoV genome. Clusters A and C are found predominantly in the Americas and Europe, while cluster C is found across East Asia (18).

## INDIVIDUAL GENETIC PREDISPOSITION/SUSCEPTIBILITY

Throughout the progression of the COVID-19 pandemic, it is clear that not all infected patients are created equal. The diversity in symptoms, morbidity, genetics, age, and geographic location all play distinct roles in viral transmission. Understanding the genetic implications underlying severe COVID-19 infection requires complex biochemical and immunological studies. Previously identified immune-related genetic variants known to be associated with susceptibility to SARS-CoV (61, 85), including mannose-binding lectin, basigin (CD147), C-C motif chemokine ligand 2 (CCL2), interleukin-12 and human leukocyte antigen (HLA) genes, might show promise due to the shared homology of the two viral genomes (41, 69, 73, 78). Utilizing our current understanding of viral entry and pathophysiology in relation to viral infection has prompted research focused on host genetic factors that may help to mitigate differences in viral replication and the innate and adaptive immune responses triggered during viral infection (75). While angiotensin-converting enzyme-2 (ACE2) receptor expression seems promising as a genetic element that could relate to immunity, no polymorphisms or mutations in ACE2 related to spike protein binding resistance have been reported in populations (8). Although rare, ACE2 variants have been identified that alter the interaction between host cells and SARS-CoV-2 causing reduced affinity of SARS-CoV-2 binding (66). Along this same line of reasoning, the gene encoding the transmembrane serine protease 2 (TMPRSS2) protease responsible for spike protein priming for viral entry has received much attention. Cell lines expressing high amounts of TMPRSS2 are highly susceptible to SARS-CoV-2 infection (43). In addition, it is known that TMPRSS2 has 2 isoforms 1 with and 1 without a 37 amino acid long cytoplasmic tail, which is thought to interact with viral spike proteins and promote viral spreading within the host (90).

Monoclonal antibodies against the spike protein of COVID-19 could play a pivotal role in blocking the virus attachment, fusion, and entry into host cells (67, 72). Antibodies against the receptor-binding domain (RBD) of the spike protein or antibodies that bind to the ACE2 receptor have been discussed as potential therapeutics (67, 72). Furthermore, recombinant RBD proteins have been shown to strongly bind to the ACE2 receptor in human and bat cells (67). There are also studies targeting glycocalyx loss as a therapeutic target of the spike protein. Importantly, blocking these initial steps in viral entry and replication could block the downstream cascade of COVID-19 pathophysiology. This would effectively decrease the morality rate of the current pandemic as it reduces the viral load in patients. Additionally, these antibodies could be potential candidates for COVID-19 antiviral and vaccine development (67). However, this therapeutic method would have very little impact on the case rate or the infectious propensity of the virus.

In addition, genetic alterations in immune response elements will be important in identifying possible gene candidates that could control host inflammatory responses that elicit the cytokine storm to help reduce secondary complications of infection by altering expression and activity of cytokines like IL-1, IL-6, interferons, and others (10). HLA is known to be one of the most polymorphic antigen systems in the body. In silico studies point out that all known HLA genotypes A, B, and C have affinity to bind SARS-CoV-2 peptides (50). Furthermore, predictive alleles have been found to have a binding capability that can infer susceptibility or possibly impart some T-cell-based immune response (50). Further studies have reviewed the genetic association of COVID-19 infection based on blood type (47) and sex, with the number of X chromosomes having an effect on susceptibility and progression of infection (20).

## COVID-19 PATHOPHYSIOLOGY

The novel coronavirus SARS-CoV-2 was originally identified as the causative agent for a series of atypical respiratory diseases in the Hubei Province of Wuhan, China in December of 2019. The disease SARS-CoV-2, which will be termed COVID-19 from herein, was officially declared a pandemic by the World Health Organization (WHO) on March 11, 2020 (82b). According to the WHO, there are 28,637,952 positive COVID-19 cases and 917,417 deaths worldwide as of September 14th, 2020 (82a). As shown in Table 1, the United States had 6,571,867 total cases resulting in 195,053 deaths, as of September 16th, 2020 according to the Centers of Disease Control and Prevention (10b). Highly populated states like California, Texas, Florida, and New York have the highest total number of cases exceeding 400,000, while less populated rural states such as Vermont, Wyoming, and Maine have total case numbers below 5,000 (10b). This reflects the predilection of the virus for more densely populated areas, allowing for higher rates of transmission in crowded areas compared with rural communities that are less densely populated. This can be seen in New York wherein the number of total deaths was 32,765 out numbering both California’s and Texas’s total deaths at 28,794 (Table 1).

**Table 1.** United States SARS-CoV-2 Statistics

Location	Total Cases	Total Deaths
Globally*	28,637,952*	917,417*
United States	6,571,867	195,053
California	760,013	14,451
Texas	668,746	14,343
Florida	660,946	12,787
New York	446,888	32,765
Race/Ethnicity		
** **Hispanic/Latino	735,892	19,340
** **American Indian	29,310	911
** **Asian, Non-Hispanic	84,055	5,792
** **Black, Non-Hispanic	449,814	24,193
** **Native Hawaiian/other Pacific Islander, non-Hispanic	9,189	201
** **White, non-Hispanic	1,016,212	59,608
** **Multiple/Other, non-Hispanic	110,112	4,930
Sex		
** **Female	2,453,649	63,820–63,829
** **Male	2,289,355	75,030–75,039
Age		
** **0–4 yr	82,351	33
** **5–17 yr	307,948	50
** **18–29 yr	1,094,403	732
** **30–39 yr	793,354	1,875
** **40–49 yr	727,519	4,508
** **50–64 yr	979,964	21,911
** **65–74 yr	358,154	29,516
** **75–84 yr	205,552	36,975
** **85 yr or older	155,295	44,438

* Updated as of 09/09/2020 (82a) and all others updated as of 09/16/2020 (10a–10c).

The epidemiology of COVID-19 to date has been found to have disproportionate impacts on populations depending on sex and ethnicity. Table 1 highlights the differences in total cases and mortality by ethnicity, sex, and age. For example, in the United States ∼51.7% of total COVID-19 cases are female and 48.3% are male (10a). In contrast, 54% of the total deaths in the United States are male compared with 46% female (10a). The most significant predictor of poor outcome and mortality associated with COVID-19 is age. The mortality data in Table 1 include available data in nine different age brackets spanning 0–85 yr and above. Most notably, patients 50 yr and above in the United States have the highest mortality rates accounting for >94% of the total deaths due to COVID-19 (Table 1; 10b, 10c). In contrast, individuals 18–29 yr old have the highest percentage of total cases at 23.3% but only have a mortality rate of ∼0.5% (10b, 10c). Older adults have higher rates of chronic health conditions that have been associated with poorer COVID-19 outcomes including hypertension, diabetes, coronary artery disease, and chronic kidney disease (62). These conditions place adults over 60 yr old at the highest risk of developing a complicated COVID-19 infection and mortality compared with younger cohorts without these conditions (62). Many patients with these conditions also take daily medications that interfere with the renin-angiotensin-aldosterone system (RAAS) such as angiotensin-converting enzyme (ACE) inhibitors for hypertension. This system has been implicated in COVID-19 infection and the virus’s ability to attach to host cells, causing dysregulated host cell responses, which subsequently results in worse outcomes (20, 25, 66).

Patients with COVID-19 often present with an array of symptoms that are similar to influenza that can make it difficult to diagnose. An epidemiological study of the first 41 patients infected with COVID-19 in Wuhan, China found that fatigue, cough, and fever were the most commonly reported symptoms (28, 31). As a result, the general symptoms of COVID-19 are challenging to diagnose without reliable testing. Positive COVID-19 classifications include the following: asymptomatic, mild, moderate, severe, and critical. Asymptomatic patients test positive and exhibit no clinical symptoms while mild cases present with acute symptoms of respiratory tract infection and digestive complications. Moderate patients experience pneumonia, without noticeable hypoxemia, with lesions on chest computerized tomography (CT) scan. Severe patients experience pneumonia with detectable hypoxemia and CT lesions while critical patients experience acute respiratory distress syndrome (ARDS) along with possible shock, encephalopathy, myocardial injury, coagulation dysfunction, heart failure, and acute kidney injury (86). In a study of 80 patients hospitalized for COVID-19, over 90% had detectable ground glass opacities present on CT scan (31, 84). A correlation was also found with the degree of inflammation seen on chest CT and lymphopenia (low white blood cell count), days of symptoms, and fever (84). Although these symptoms are often informative in diagnosis, COVID-19 has an unpredictable clinical course. As a result, 13.8% of positive patients had severe cases that required an in-patient hospital stay, with 4.7% requiring intensive care unit hospitalization and 2.3% of cases resulting in death (31). Taken together, these factors make COVID-19 difficult to manage and hard for clinicians to diagnose and predict clinical outcomes. Furthermore, real-time generation of data using artificial intelligence is an absolute priority to combat the spread, diagnosis, treatment, and categorized susceptibility to COVID-19 (1).

**Fig. 1.**
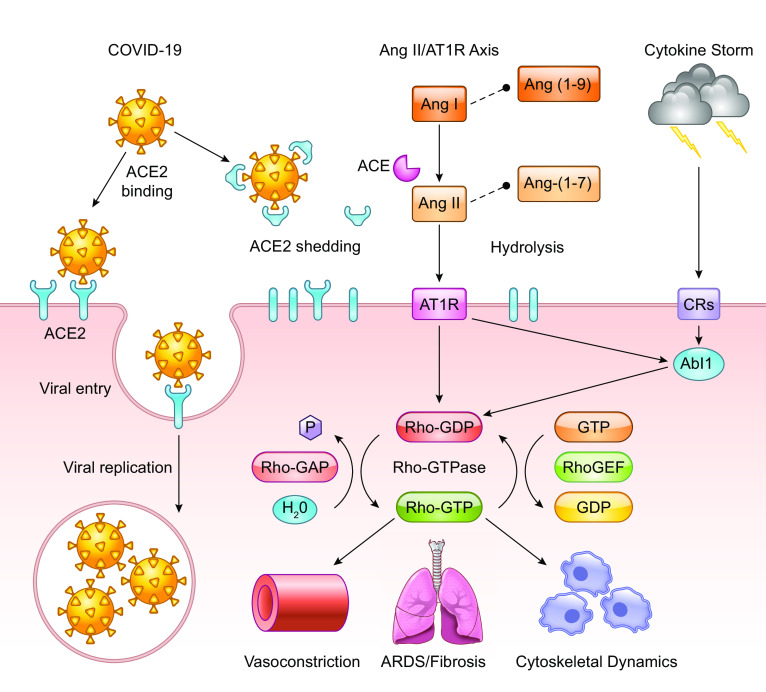
Biological effects of COVID-19 infection on angiotensin-converting enzyme 2 (ACE2) receptor and GTPase signaling pathways. The COVID-19 virus can bind and interact with both shed ACE2 and the cell membrane-bound ACE2 receptor. COVID-19 particles utilize and degrade membrane bound ACE2 receptors to gain entry into host cells. Virus particles also bind shed ACE2 causing a reduction in free ACE2 thus preventing the hydrolysis of ANG I/II into ANG-(1-9)/ANG-(1-7), which results in an imbalanced renin-angiotensin system that becomes skewed toward the ANG II/angiotensin type 1 receptor (AT_1_R) axis. COVID-19 produces an inflammatory response, i.e., the cytokine storm, which triggers cellular activation through cytokine receptors (CRs). Upon infection, these interactions favor detrimental complications such as acute respiratory distress syndrome (ARDS)/pulmonary fibrosis, vasoconstriction and alters cytoskeletal dynamics including cell proliferation, migration, and cytoskeletal composition. Intracellular elements such as Abelson murine leukemia viral oncogene homolog 1 kinase and Rho GTPase-associated proteins play a significant role in controlling polymerization of F-actin, maintaining the density of the extracellular matrix (ECM), and modulating myofibroblast proliferation, and the development of pulmonary fibrosis.

Understanding the pathophysiology of COVID-19 is critical to improving patient outcomes and determining how we can overcome the current pandemic. A key component to the virus being able to enter host cells and replicate is the ACE2 receptor, which is highly expressed in alveolar epithelial cells of the lung as confirmed by RNA-seq (91). The viral glycoprotein spike casing found on the exterior of a virus particle is responsible for eliciting viral entry into susceptible host cells (27). The process of viral entry requires priming of the spike protein by host expressed TMPRSS2, which interacts with the spike protein and cleaves it into two functional subunits known as S1 and S2 (27, 43, 66). The S1 subunit directly interacts with the ACE2 receptor, leaving the S2 subunit to facilitate viral fusion with the host cell membrane (Fig. 1; 25, 27, 41a). Internalization and replication of virus subsequently cause degradation of membrane-bound ACE2 receptors (27), which in turn causes an increase in angiotensin II (ANG II) and the angiotensin type 1 receptor (AT_1_R) (Fig. 1). Angiotensinogen is cleaved by renin to angiotensin I (ANG I). ANG I is cleaved via ACE to ANG II, wherein it can freely interact with AT_1_R and angiotensin type 2 receptor (AT_2_R). Excess ANG I and II are hydrolyzed by ACE2 to become the heptapeptides ANG-(1-9)/ANG-(1-7) (Fig. 1). Reduced or bound ACE2 is unable to hydrolyze ANG I/II, which results in an inability of the counterbalancing effects of the Mas receptor (Mas-R) to protect against detrimental disease/immune complications. As a result of COVID-19 infection, decreases in ACE2 cause elevated activity in the ANG II/AT_1_R axis, resulting in an inflammatory immune response (76). This deficiency leads to many adverse outcomes for patients including interstitial fibrosis, myocardial hypertrophy, endothelial fibrosis, and increased inflammation (76). Additionally, thrombosis and hypercoagulation secondary to platelet activation after lung epithelial damage are seen in patients with severe infections (39, 86). Further consequences of hypercoagulation include disseminated intravascular coagulation, pulmonary embolisms, cardiac complications, and an increased risk of death (39, 70). Coagulation is induced as a protective physiological control in response to vascular leak but in turn elicits dangerous consequences in COVID-19 patients. Often the physiologic response mechanisms to vascular leak and permeability fail, which allows for enhanced viral invasion, thus amplifying the problem in host cells on two separate fronts (86).

## CARDIOVASCULAR DISEASE AND COVID-19

The highest risk factors for severe COVID-19 infection, including ARDS, is diabetes, hypertension, and a history of heart disease (76). Although the primary target of COVID-19 is the lungs, it can also have detrimental effects on the cardiovascular system. These comorbidities result in an upregulation of ACE2 on the cell surface of perivascular pericytes and cardiomyocytes, which is exploited by COVID-19 as the route of entry and infection (25). The leading cause of death in the United States is cardiovascular disease (CVD) causing more than 800,000 deaths in 2016 (20a). A meta-analysis study in China found that COVID-19 causes acute cardiac injury in roughly 8.0% of patients, which poses concern for those that have a preexisting cardiac or metabolic condition (40). Cardiac injury may present as common arrhythmias, myocarditis, cardiogenic shock, and/or heart failure (24, 49). Patients with prior cardiac history, including acute coronary syndrome and angina or myocardial infraction, have a higher risk for developing pneumonia and a decreased cardiac reserve that poses significant risks if they contract COVID-19 (40, 88). The middle east respiratory syndrome coronavirus (MERS-CoV) is in the same corona virus family as COVID-19, has similar clinical outcomes, and has been extensively studied in patients with these comorbid conditions (5). In an analysis of 637 MERS-CoV patient cases, 30% had cardiac diseases and 50% had hypertension or diabetes (5, 88). These cardiovascular disorders are highly prevalent in the United States, placing this vulnerable population in a higher risk category for acquiring severe infection with COVID-19. Patients with CVD may not have the ability to maintain cardiovascular function upon COVID-19 infection, leading to an increase in metabolic demand, exacerbating cardiovascular conditions thus increasing their risk for severe outcomes (68).

## COVID-19 AND ACUTE RESPIRATORY DISTRESS SYNDROME

The host immune response to COVID-19 is similar to ARDS and therefore treatment modalities may be beneficial in treating COVID-19 patients. ARDS is defined clinically as bilateral neutrophilic infiltrates seen on imaging, acute hypoxia, and pulmonary edema (19, 30). ARDS is caused by a dysregulated immune response with a fibroproliferative component due to excessive levels of cytokines, chemokines, and reactive oxygen species (30). ARDS-positive patients exhibit elevated levels of proinflammatory cytokines including IFN-y, IL-6, IL-12, and IL-1 compared with patients with uncomplicated COVID-19 infections (12). A study in ARDS positive mice confirmed these findings, wherein bronchoalveolar lavage fluid from ARDS positive mice strains had higher levels of TNF-α, IL-6, and vascular endothelial growth factor (VEGF) with reduced levels of IL-10 in comparison with controls (57). Similarly, patients hospitalized with severe COVID-19 infections have elevated cytokine profiles that are reflective of what defines a “cytokine storm.” The cytokine storm is a result of an uncontrolled immune response due to systemic inflammation and hemodynamic instability due to the abundance of proinflammatory cytokines that include IL-1, IL-6, IL-18, IFN-γ, and TNF-α (Fig. 1) (65). As a result, new therapies are needed to thwart the immune response including nonconventional immunomodulation (22) to control the increase in proinflammatory cytokines that results in an accumulation of macrophages, neutrophils, and T cells from the circulation to the lung destroying the cell-cell interactions resulting in severe cases of ARDS. These findings suggest that patients suffering from ARDS and severe COVID-19 have a failed anti-inflammatory response that contributes to the excessive inflammatory damage caused by a host of proinflammatory cytokines wreaking havoc on lung tissue (58). Extensive damage to epithelial and endothelial cells of the lung triggers apoptotic destruction (12) leading to changes in the cellular junctions in alveolar tissue, thus increasing vascular permeability and ultimately alveolar fluid leak (30). Consequently, these cellular changes result in the pulmonary edema classically seen in ARDS patients (30), which is further complicated by an increase in dysregulated epithelial cell remodeling contributing to pulmonary fibrosis (12), a common cause of mortality in ARDS patients (30).

## ABL1 AND VASCULAR PERMEABILITY

Abelson murine leukemia viral oncogene homolog 1 (Abl1) is a widely expressed nonreceptor tyrosine kinase that has been implicated in controlling cell morphology, growth, and survival (79, 82). Abl1 is activated through a variety of receptor interactions and factors including cytokines, DNA damage, and oxidative stress (77). Abl1 plays a major role in modulating cytoskeletal dynamics influencing cell proliferation, cell survival, endocytosis, membrane trafficking, and cell-cell junctions and is also implicated in solid tumor proliferation and survival (34). Abl1 signals proteins that are critical to extracellular matrix (ECM) function and composition including the formation of actin stress fibers. These fibers interact with F-actin, inducing filopodia, which can alter cell-cell junctions (59, 79, 82).

Inhibition of Abl1 leads to increased Rho-Rock signaling, actomyosin contractility, and destabilization of cell-cell adhesions leading to an increase in barrier disruption (16, 59, 87). There is a direct implication of Abl1 as a therapeutic target to regulate GTPases in an effort to control ARDS and fibrosis as a result of disrupted endothelial barrier function and vascular leak in the lungs of ARDS patients (45, 82, 87). This critical association can be detrimental in ARDS, pulmonary fibrosis, and in severe cases of COVID-19 infection when vascular leak becomes uncontrolled and leads to sepsis (30). Multiple studies have investigated therapies to preserve endothelial barrier function. This includes the therapeutic use of low molecular weight heparin to combat the degradation of heparin sulfate by heparinase, thus protecting the endothelial barrier (7). Furthermore, the drug imatinib, an Abl1 inhibitor, has been investigated for possible repurposing and use for lung injury patients (36, 82). One study found that pretreatment with imatinib protected against acute lung injury in mice (36) and may have potential to be repurposed in patients suffering from ARDS and/or COVID-19. Case studies report that imatinib resolved pneumonitis and pulmonary fibrosis secondary to antibiotics (9, 59). Selective targeting of Abl1-based therapeutics needs further investigation to avoid potential negative side effects. For example, studies have shown that inhibiting Abl1 leads to increased endothelial permeability because of F-actin alternations and is amplified in cells undergoing cyclic stretch secondary to mechanical ventilation (38, 59). As a result, increased vascular permeability will lead to an acceleration in vascular leak, exacerbating outcomes in ARDS patients.

## PULMONARY FIBROSIS AND GTPase SIGNALING

While much is known about the progression of COVID-19 and ARDS, the mechanism of pathophysiology and associated treatment strategies are still under investigation. One such area includes GTPase signaling and its role in the development of ARDS and subsequent pulmonary fibrosis. Pulmonary fibrosis is caused by excessive fibroblasts and ECM protein deposits in the lungs, referred to as scarring of the lungs (4). Myofibroblasts are derived from resident fibroblasts and mesenchymal cells in the lung that express high amounts of smooth muscle actin (29) and are major players in the production of excess collagen leading to progressive fibrosis in patients (6). The overall ECM composition and stiffness have a direct impact on the degree of fibroblast migration, proliferation, and differentiation (4). Studies have shown that denser ECM substrates in later stages of disease show higher fibroblast migration levels compared with decreased fibroblasts migration in less stiff substrates as seen in earlier stages (6). One pathway with therapeutic implications in these physiological processes is the Rho GTPase signaling cascade (6, 82).

Rho GTPase signaling has vast cellular implications in the control of actin and myosin stress fiber formation, regulation of cell adhesion molecules, cell migration, and common cellular functions (81). In addition, Rho GTPases play significant roles in cytoskeletal actin remodeling by polymerization and de-polymerization of monomeric G-actin leading to the conversion of F-actin (29). Increases in F-actin fibers causes stiffening of the ECM in patients suffering from ARDS leading to decreased vascular compliance (33). ARDS patients often require some form of oxygen supplementation due to severe hypoxemia. These measures often lead to hyperoxia and cause acute lung injury compounding damage to the lungs (30, 44). Interestingly, hyperoxia in mice was found to activate the Rho/ROCK GTPase pathway and led to an increase in cell stiffness secondary to F-actin increase. However, when these mice were treated with Y-27632, a Rho inhibitor, the cytoskeletal changes in stiffness were prevented (81). These results suggest a possible connection in the control of GTPase signaling and ARDS and/or fibrosis complications seen in patients who require supplemental oxygen. Therefore, therapeutically modulating the increased activity of the GTPase cascade could decrease the adverse effects of ARDS pathogenesis secondary to ECM remodeling events.

As previously discussed, the Rho GTPase pathway regulates ECM density (81, 82). This leads to the conclusion that higher activation levels of Rho and associated downstream targets lead to a higher levels of fibroblast proliferation. The ACE2 cascade is protective against lung fibrosis through activation of Rho GTPase pathways, while ACE is damaging and stimulates fibrosis in lung endothelial cells (46). These findings correlate to the virus’s predilection for patients with a history of obesity, hypertension, and CVD as these chronic conditions have been found to have lower levels of ACE2 at baseline (76). Therefore, the interplay between the ACE2/ACE and the Rho GTPase pathway may be an important association that could be a target for therapeutics to block lung fibrosis that results in ARDS and a majority of the mortality in COVID-19 patients. A study performed by Haung et al. proved this association by showing blockade of the Rho GTPase pathway inhibits matrix stiffness and alters stress fiber formation in fibroblasts (29). Therefore, Rho is actively involved in the underlying mechanism of pulmonary fibrosis by controlling proteins critical to modulating the ECM. A few significant trials have tested this theory for idiopathic pulmonary fibrosis by using nintedanib, a multikinase inhibitor, and pirfenidone, a small molecule antifibrotic, both of which were shown to reduce loss of lung functioning in pulmonary fibrosis patients (6).

## STRATEGIES FOR SARS-CoV-2 THERAPEUTICS

Controlling the extensive spread and progression of SARS-CoV-2 has proven very difficult and will require a multidisciplinary approach with global collaboration. While certain areas of interest in SARS-CoV-2 remain unknown, past coronavirus knowledge provides scientists with the foundation for the development and/or repurposing of therapeutic interventions and vaccine development. Since the spike protein of each individual type of coronavirus is unique, this protein is currently being targeted in vaccine development as an approach to block initial entry of the virus (2, 63). Multiple vaccines have entered clinical trials, the first of which is an RNA-based vaccine, mRNA-1273 (26). This vaccine entered phase I clinical trials on March 16, 2020 in collaboration with the National Institutes of Health (NIH), utilizing 45 healthy participants ranging in ages from 18 to 55 yr old (2). Although science has provided the foundational studies on vaccine development, the time needed to assess the safety and efficacy of vaccine candidates is a major bottleneck in the overall process.

While vaccines are being tested and manufactured, novel therapeutic treatments for the control and clinical management of COVID-19 infection are needed. Numerous approaches for treatment have been anecdotally reviewed in mainstream media; however, there are currently no Food and Drug Administration-approved medications for the treatment of COVID-19 infections (13). Still, there are a number of medications under evaluation for their effectiveness as potential antivirals that are recommended for use in the National Institutes of Health COVID-19 treatment guidelines (13). A noteworthy example of a current therapeutic intervention includes the use of convalescent plasma therapy (15, 64). In this process, plasma-containing-neutralizing antibodies, removed from a donor who has previously recovered from a SARS-CoV-2 infection, are administered to infected patients to impart protection. Another unique therapeutic method involves treatment with soluble recombinant human ACE2 to disrupt viral entry via the spike protein-ACE2 interaction. Initial testing with recombinant ACE2 in simian cell lines and engineered human tissues shows promise in reducing viral load in a dose-dependent fashion (48). Finally, due to the high sense of urgency in clinical treatment of COVID-19 infection, the repurposing of known antiviral drugs has been explored with extreme caution, and the rationale are outlined in the NIH COVID-19 Treatment Guidelines (13). The treatment guidelines have the current recommendations either for or against the use of known antiviral drugs and the existing clinical trial data from the National Institutes of Health (13). Furthermore, implications for the use of some drugs have been identified using in silico databases that predict protein-protein interactions (23, 74). Antiviral therapies contained in these studies include remdesivir, ivermectin, favipiravir, kaletra, and chloroquine/hydroxychloroquine with or without azithromycin (13, 23, 74).

## CONCLUSION

The COVID-19 pandemic continues to pose a serious public health threat to nations around the world, as effective antiviral therapeutics or vaccines are yet to be developed. The primary goal in the COVID-19 pandemic is to limit transmission and define clinical management that improves the cure rate and effectively reduces the overall mortality rate. To achieve this goal, a complete understanding of all aspects of coronaviruses is needed to prevent or lessen their threat to society in the future. A thorough understanding of the epidemiology, pathophysiology and pandemic response efforts to combat COVID-19 is an invaluable lesson to society providing a protocol to fight future pandemics should they occur. Most importantly, scientific insights gained in the fight against COVID-19 will provide the evidence needed to develop vaccines and antiviral therapeutics that target viral entry, immune response and activation, and clinical management of secondary complications associated with severe infections.

## GRANTS

M.P.M. and A.L.N-K. acknowledge funding support from the University of Toledo University Research Funding Opportunities (URFO) Program - Interdisciplinary Research Initiation Award I-127366-01.

## DISCLOSURES

No conflicts of interest, financial or otherwise, are declared by the authors.

## AUTHOR CONTRIBUTIONS

C.A.P., M.P.M., and A.L.N-K. prepared figures; C.A.P., M.P.M., and A.L.N-K. drafted manuscript; C.A.P., M.P.M., and A.L.N-K. edited and revised manuscript; C.A.P., M.P.M., and A.L.N-K. approved final version of manuscript.
